# Supplementary material for: Use of Stromal Intervention and Exogenous Neoantigen Vaccination to Boost Pancreatic Cancer Chemo-Immunotherapy by Nanocarriers
Source: Bioengineering (Basel). 2023 Oct 16;10(10):1205. doi: 10.3390/bioengineering10101205 (PMC10604647; doi:10.3390/bioengineering10101205)
Supplement: Supplementary file 1 [file bioengineering-10-01205-s001.zip › bioengineering-2589551-supplementary.pdf]

Supporting Information

Use of Stromal Intervention and Exogenous Neoantigen Vaccination to Boost Pancreatic Cancer Chemo-Immunotherapy by Nanocarriers

Saborni Chattopadhyay[1], Yu-Pei Liao[1,2], Xiang Wang[1,2] and Andre E. Nel[1,2,3†]*

[†]Professor of Medicine in the David Geffen School of Medicine at UCLA and Research Director of the California NanoSystems Institute at UCLA, Los Angeles, California- 90095, United States
[1]California NanoSystems Institute, University of California, Los Angeles, California- 90095, United States
[2]Division of NanoMedicine, Department of Medicine, David Geffen School of Medicine, University of California, Los Angeles, California- 90095, United States
[3]Jonsson Comprehensive Cancer Center, University of California, Los Angeles, California- 90095, United States

ORCID ID:
Saborni Chattopadhyay: 0000-0002-9143-0107
Yu-Pei Liao: 0000-0001-7239-9426
Xiang Wang: 0000- 0000-0002-6647-0684
André Nel: 0000-0002-5232-4686

**Correspondence should be addressed to:*
André E. Nel,
Division of NanoMedicine, Department of Medicine, University of California, Los Angeles,
52-175 CHS, Los Angeles, California 90095, USA.
Phone: 310.825.6620;
E-mail: anel@mednet.ucla.edu

Table of Contents:

Figure #	Title	Page#
S1	***In vivo*** **therapeutic efficacy of liposomal LXA4 irinotecan in subcutaneous KPC tumor model.**	3
S2	**Analysis of immune infiltration in tumors following liposomal LXA4 irinotecan treatment in a subcutaneous KPC pancreatic cancer model.**	5
S3	**Analysis of immune infiltration in subcutaneous pancreatic tumors from vaccinated mice.**	7
S4	**Analysis of immune infiltration in orthotopic KPC-luc tumors.**	9

A

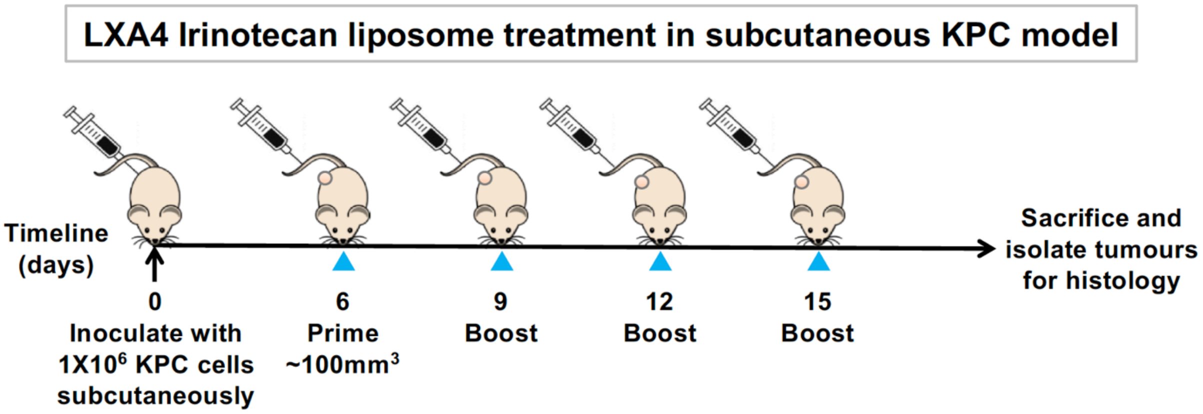

B

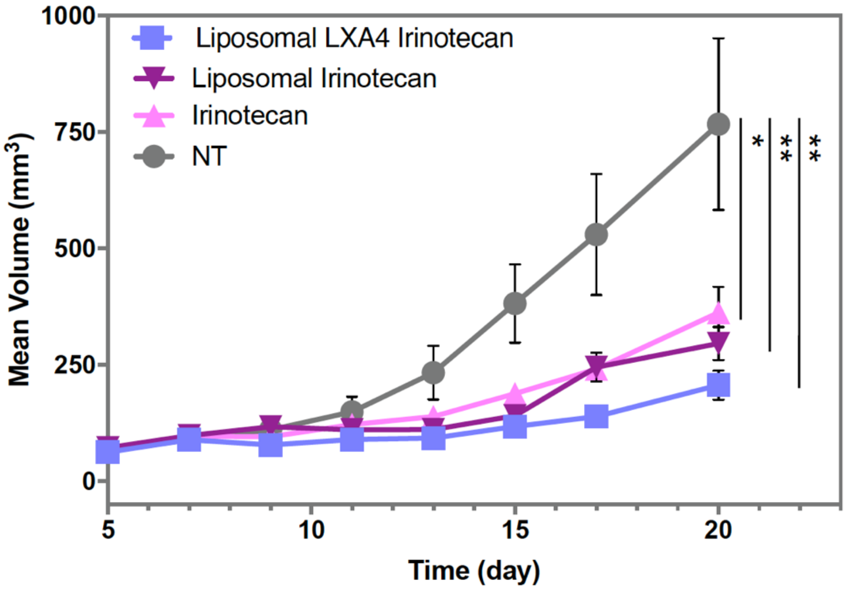

C

Bilayer composition of liposomes				
**Lipids**	**DSPC**	**Cholesterol**	**DSPE-PEG$_{2kDa}$**	**LXA4**
**Units**	**(Mol%)**	**(Mol%)**	**(Mol%)**	**(Mol%)**
Lipo-Irinotecan	55.8	39	5	--
Lipo-LXA4 Irinotecan	55.43	39.6	4.7	0.2

Figure S1. *In vivo* therapeutic efficacy of liposomal LXA4 irinotecan in a subcutaneous KPC tumor model. (A) Female B6129SF1/J mice (7 weeks old) were used to assess the therapeutic effects of a liposome that encapsulates LXA4 and irinotecan. The animals were injected subcutaneously with 1x10^6 viable KPC cells (in a 1:1 mixture of PBS and Matrigel) on the left hind flank. Six days later, mice with palpable growing tumors were randomly assigned to treatment groups, comprised of 5 mice per group. These tumor-bearing mice were injected with either free or

liposomal irinotecan (40mg/kg) or the LXA4/irinotecan liposome, delivering the same amount of irinotecan plus 90μg/kg LXA4, twice a week, for a total 4 injections. Mice were weighed and tumor size were measured with digital calipers every 3-4 days. Mice were sacrificed after 21 days. (B) Tumor volume was calculated according to the formula $V(mm^3) = 0.52 \times (\text{length in mm}) \times (\text{width in mm})^2$. (C) Table to show the bilayer composition of the synthesized LXA4-irinotecan liposomes, which are described in Figure 2.

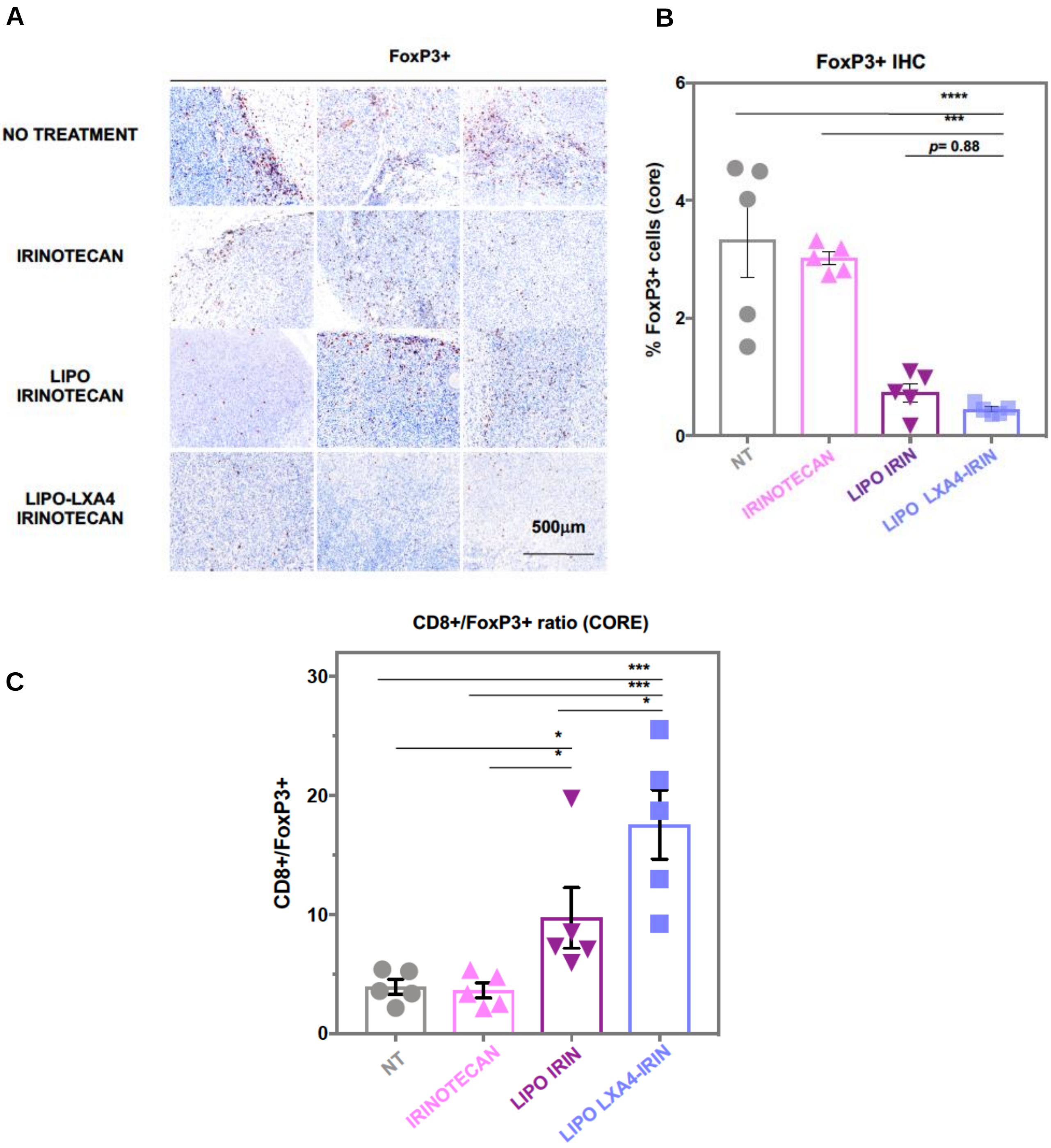
A
FoxP3+
NO TREATMENT
IRINOTECAN
LIPO IRINOTECAN
LIPO-LXA4 IRINOTECAN
500µm
B
FoxP3+ IHC
% FoxP3+ cells (core)
0
2
4
6
****
***
p= 0.88
NT
IRINOTECAN
LIPO IRIN
LIPO LXA4-IRIN
C
CD8+/FoxP3+ ratio (CORE)
CD8+/FoxP3+
0
10
20
30
***
***
*
*
*
NT
IRINOTECAN
LIPO IRIN
LIPO LXA4-IRIN

D

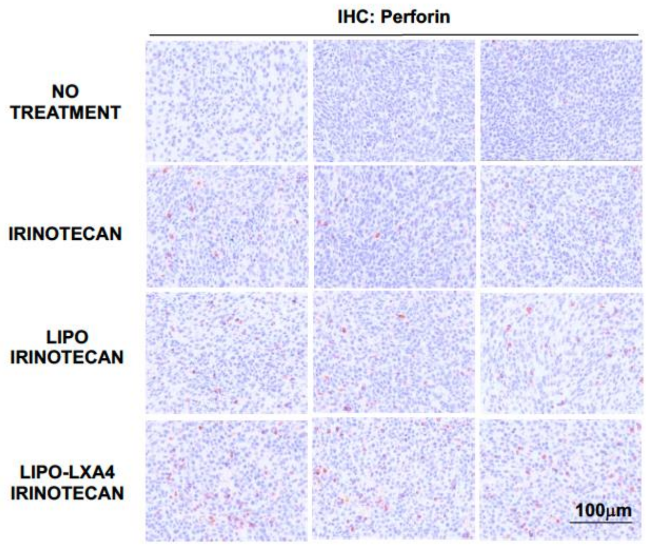

E

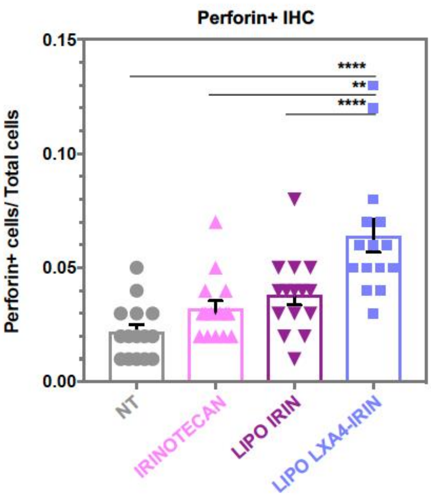

Figure S2: Immunohistochemistry analysis of the tumors used, to perform the subcutaneous experiment described in Figure S1 and Figure 3 in the body of the manuscript. The animals were treated as described in Figure S1. After 21 days of tumor implantation, animals were sacrificed and tumors harvested and fixed in 4% formalin followed by paraffin embedding and sectioning into 5 μm sections. These sections were analysed by multicolour fluorescent imaging for CTLs and regulatory T cells, using CD8 and FoxP3 markers. (A-B) Visual IHC images and quantification of T_{regs}. The quantitative data were used for calculating the $CD8^{+}$/Foxp3 ratio, which is also displayed in Figure 3C. These data demonstrate that treatment with the dual delivery LXA4-irinotecan liposome induced a statistically significant decline in the number of Tregs, which was accompanied by the most significant increase in the $CD8^{+}$/Foxp3 ratio shown in Panel (C). (D-E) IHC analysis for perforin expression also demonstrated the statistically significant highest perforin deposition in the TME during treatment with the dual-delivery liposome compared to other treatment groups. Scale bar= 100μm

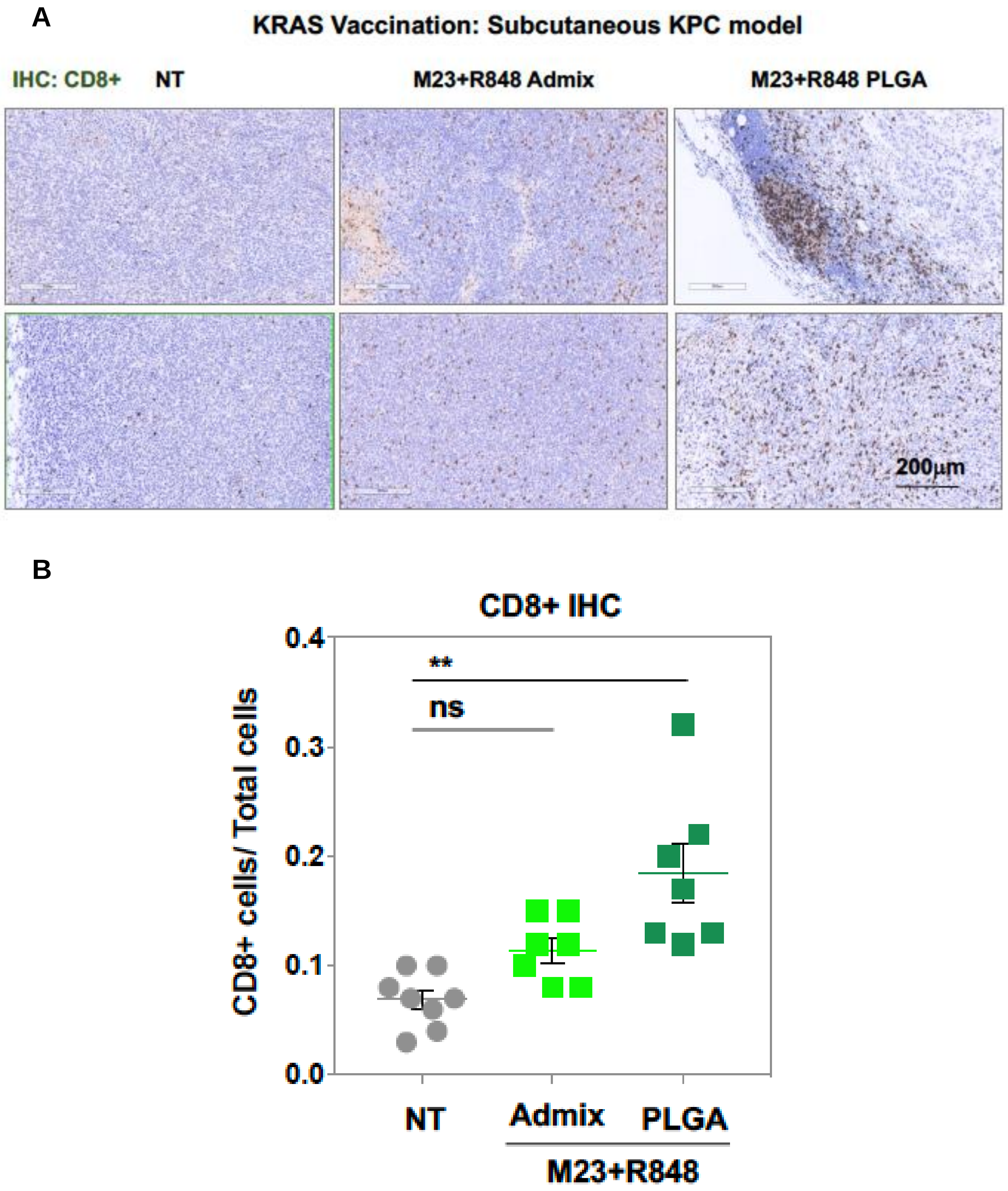
A
KRAS Vaccination: Subcutaneous KPC model
IHC: CD8+
NT
M23+R848 Admix
M23+R848 PLGA
200μm
B
CD8+ IHC
**
ns
CD8+ cells/ Total cells
0.4
0.3
0.2
0.1
0.0
NT
Admix
PLGA
M23+R848

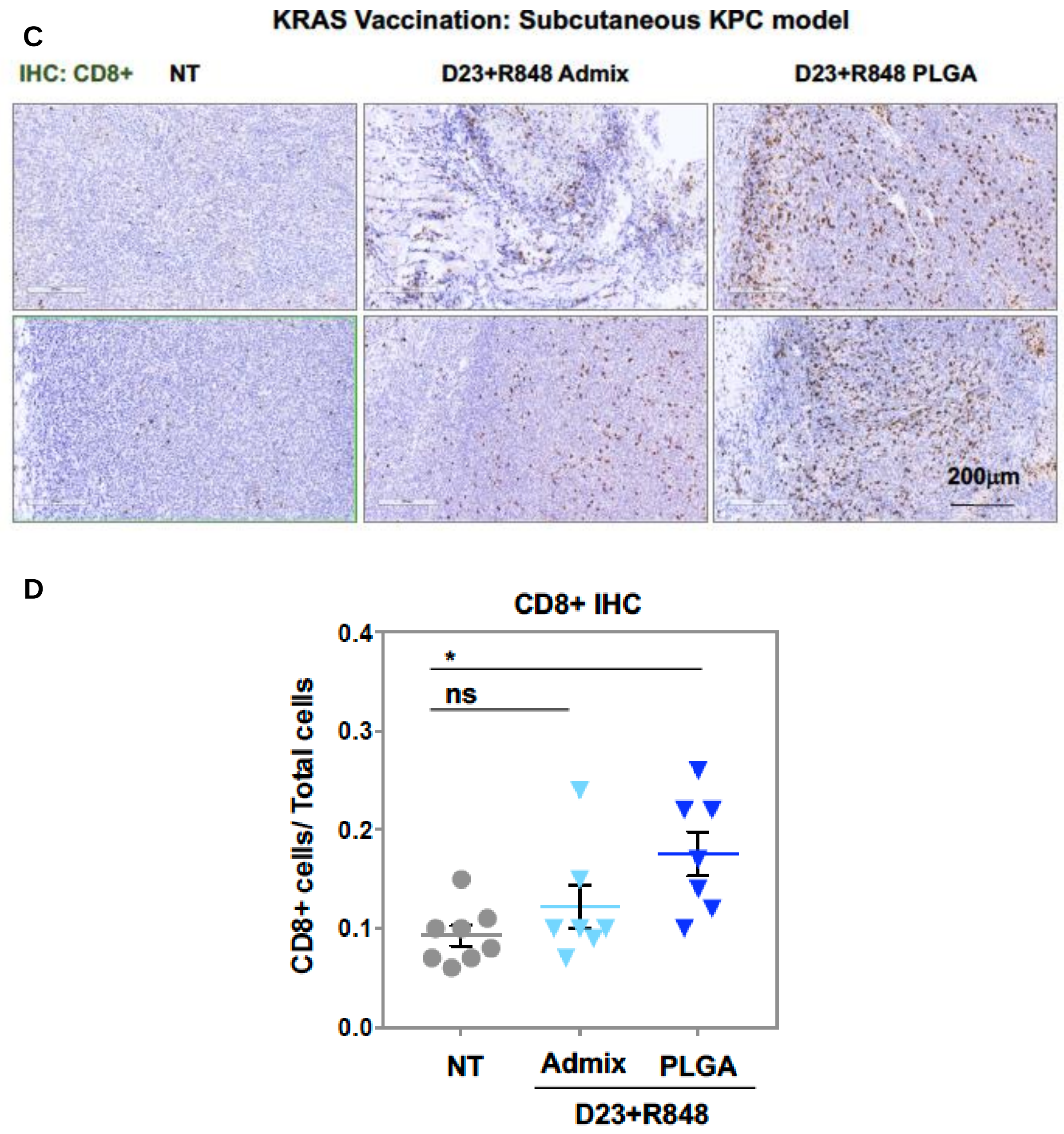

Figure S3: Analysis of immune infiltration in subcutaneous pancreatic tumors in vaccinated mice. For the subcutaneous Kras vaccine model, female B6129SF1/J mice (7 weeks old) were vaccinated subcutaneously on the right hind flank and boosted twice every 7 days with 10μg encapsulated or free peptide plus 2μg R848, as described in Figure 6. The animals were then challenged subcutaneously on the left hind flank with 0.1×10^6 viable KPC cells (in a 1:1 mixture of PBS and Matrigel), a day before the final boost. After 45 days from the onset of tumor challenge, animals were sacrificed, tumors harvested and fixed in 4% formalin before paraffin embedding. Immunohistochemistry was performed on 5μm tumor sections for $CD8^+$ T-cells, which were (A) imaged and (B) quantified. Expression was statistically significant increase for animals treated with $M23{+}R848_{PLGA}$ nanoparticles. Similarly, images showing $CD8^+$ expression (C) and quantification (D), showed a significant increase in $CD8^+$ expression in animals vaccinated with the $D23{+}R848_{PLGA}$ nanoparticles compared to

unvaccinated or animals receiving the (D23+R848) admix. ImageQuant tification was performed with ImageScope software. Scale bar= 200μm

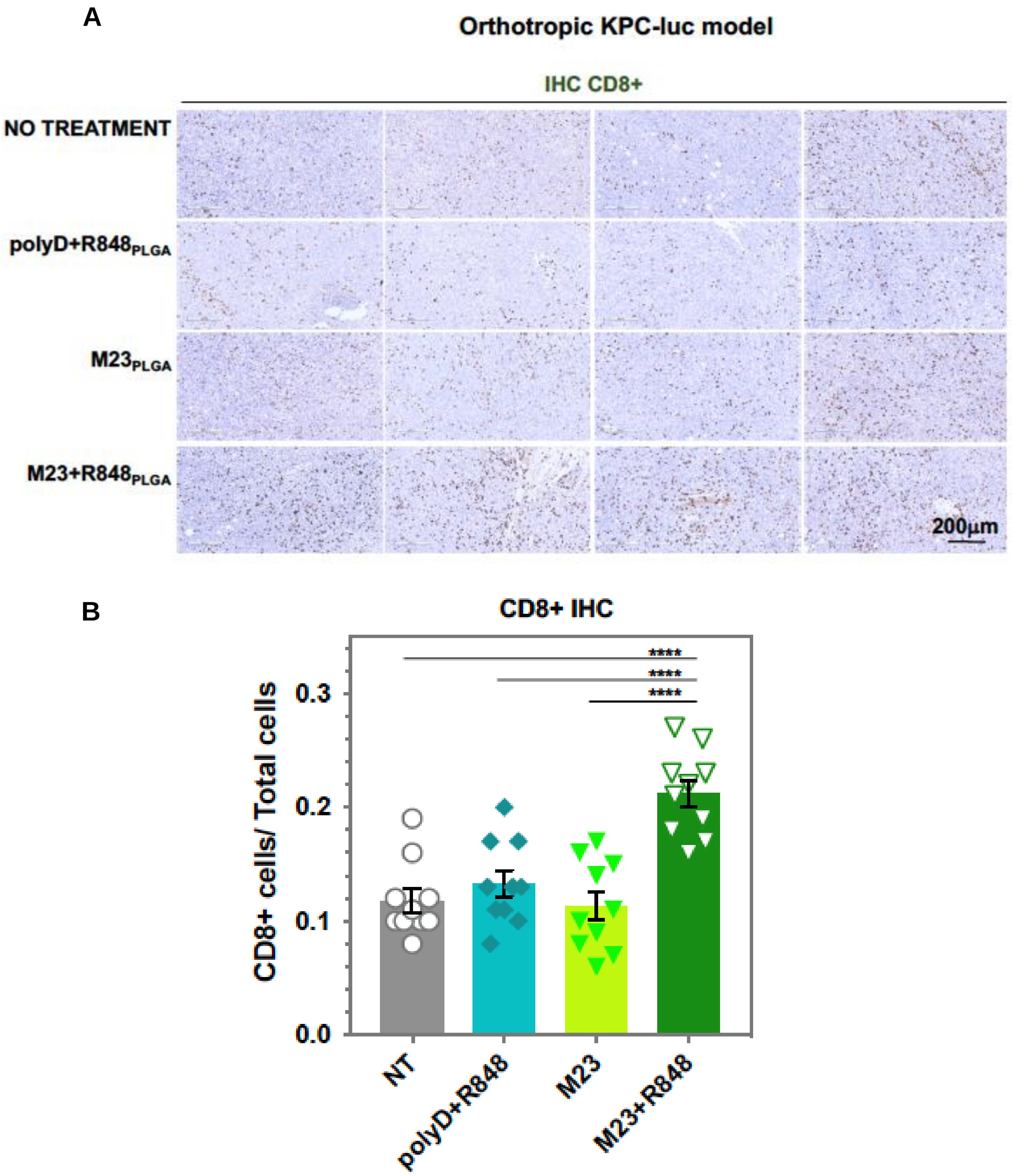

C

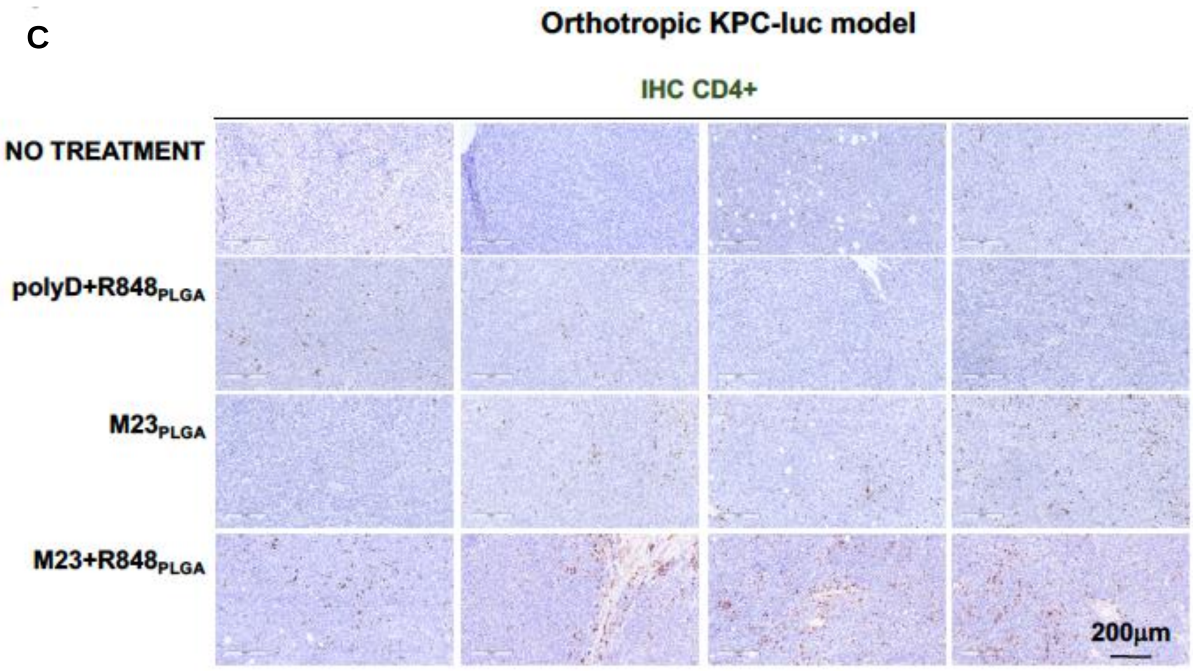

D

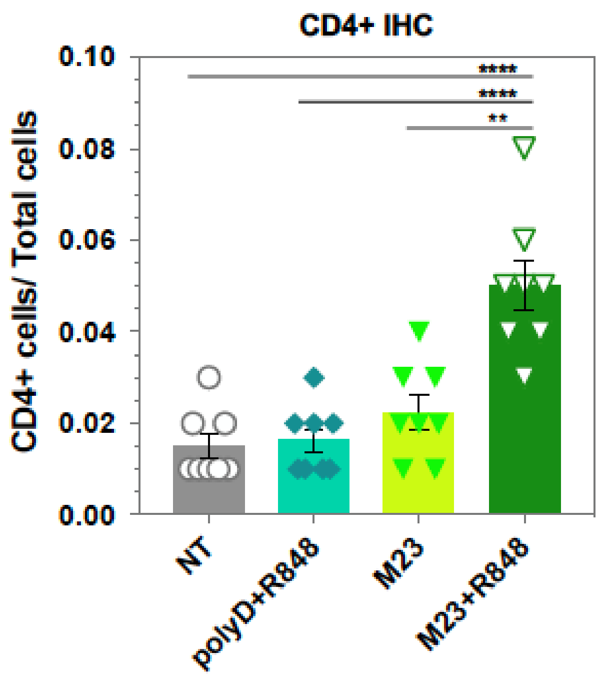

Figure S4: Analysis of immune recruitment to the tumors in animals used in the orthotopic experiment, shown in Figure 8. For the orthotopic Kras vaccine model, female B6129SF1/J mice were vaccinated subcutaneously on the right hind flank and boosted twice every 7 days with 10µg peptide M23 and 2µg R848 nanoparticles. Control groups included unvaccinated mice and animals receiving the (polyD+R848) $_{PLGA}$ or M23$_{PLGA}$ (no agonist) nanoparticles. After 13 days from the onset of vaccination, the pancreata were injected 0.5x10^{6} viable KPC luciferase cells (< 12 passages) suspended in a 1:1 PBS to Matrigel solution. Tumor-bearing mice were sacrificed on day 16 and tumors harvested. These tumors were fixed, embedded and used for preparing 5µm sections. IHC analysis of the sections were performed for the

visualization and quantification of (A-B) $CD4^+$ T-cells, (and C-D) $CD8^+$ T-cells, as described in Figure 8. Scale bar= 200μm.

Statistical analysis:

All graphs and statistical analysis were made using GraphPad Prism Vol.5 (GraphPad Software Inc., San Diego, CA). All values are expressed as a mean ± standard error of the mean (SEM). Statistical significance of the results was performed by either a two-tailed unpaired student's t-test for comparison of two treatment groups or a one-way ANOVA to compare multiple treatment groups. Differences were considered significant for a p-value of $*p < 0.05$, $**p < 0.01$, $***p < 0.001$, $****p < 0.0001$ respectively.
